# Draft Genome Sequences of Five Cystobasidium ongulense Strains Isolated from Areas near Syowa Station, East Antarctica

**DOI:** 10.1128/mra.00224-22

**Published:** 2022-06-13

**Authors:** Masaharu Tsuji, Jun-ichi Ishihara, Yasuhiro Gotoh, Tetsuya Hayashi, Hiroki Takahashi

**Affiliations:** a Department of Materials Chemistry, National Institute of Technology (KOSEN), Asahikawa College, Asahikawa, Hokkaido, Japan; b Medical Mycology Research Center, Chiba University, Chiba, Chiba, Japan; c Department of Bacteriology, Faculty of Medical Sciences, Kyushu University, Higashi-ku, Fukuoka, Japan; University of California, Riverside

## Abstract

Cystobasidium ongulense has been reported from East Ongul Island near Syowa Station, East Antarctica, as a new basidiomycetous yeast species. This species has cold active lipases and cellulases that are active even at subzero temperatures. We report draft genome sequences of five *Cystobasidium ongulense* strains isolated from East Antarctica.

## ANNOUNCEMENT

Cystobasidium ongulense was isolated from East Ongul Island, East Antarctica, and reported as a new species of basidiomycete yeast (1). Since this yeast can grow in a wide range of temperatures, from −3°C to 30°C, the genomic information of this yeast is expected to be valuable as a new genetic resource (2).

This study analyzed the whole-genome sequences of two strains (9A-2 = JCM 31527 and 9A-5 = JCM 31528) isolated from East Ongul Island and three strains (051-20-1, 056-1-20Y-3, and 056-20-8) isolated from Inhovde, near Syowa Station.

Each strain was cultured in yeast extract-peptone-dextrose (YPD) liquid medium (Difco) at 15°C for 5 days. The cells from cultures were collected by centrifugation at 3,500 × *g* for 5 min at 4°C. The cell pellets were washed with sterile distilled water and precipitated by centrifugation again. The cell pellets were dried using a vacuum freeze dryer. The lyophilized cells were powdered in a mortar and used for genome extraction. The genomic DNA of five *Cystobasidium ongulense* strains was extracted and purified using a NucleoBond AXG100 column with a Nucleo buffer set III (TaKaRa Bio) according to the manufacturer’s protocol. The concentration and purification of the genomic DNA were determined using a NanoDrop spectrophotometer (Thermo Scientific) and the Qubit double-stranded DNA (dsDNA) broad-range (BR) assay kit (Thermo Scientific). Then, the genomic DNA was digested using the microTUBE (Covaris), and the genomic library was constructed using the Illumina TruSeq DNA PCR-free library preparation kit (Illumina). The quality of the library was confirmed using the Quant-iT PicoGreen dsDNA assay kit, and the library size was checked using the Agilent 2200 TapeStation system (Agilent Technologies, Inc.). The paired-end sequence reaction was performed on a HiSeq 2500 instrument (Illumina). Adapters and low-quality bases were trimmed using fastp ver. 0.12.4 (3). Assembly of the nuclear genomes was conducted using VelvetOptimiser ver. 2.2.6 (4). The completeness of the assemblies was assessed using BUSCO ver. 5.2.2 (5) with the database basidiomycota_odb10 (the number of the marker is 1764). After the identification of repeat sequences using RepeatModeler ver. 2.0.1 and RepeatMasker ver. 4.1.2 (6), annotation was performed using the BRAKER2 ver. 2.1.6 (7–11) automated annotation pipeline using the fungi protein data set from OrthoDB ver. 10 (12). BRAKER2 *ab initio* gene predictions were carried out using AUGUSTUS ver. 3.4.0 (10) and GeneMark-EP+ ver. 4.64 (13). The resulting BRAKER2 gtf file was parsed using TSEBRA (14) and AGAT ver. 0.8.0 (15). Finally, functional annotation was performed using InterProScan ver. 5 (16), Phobius ver. 1.01 (17), antiSMASH ver. 6.0 (18), and EggNog mapper ver. 2.1.3 (19) against the eggNOG database ver. 5.0.1 (20) using diamond ver. 2.0.9 (21).

A summary of the genomic analysis results of the five *C. ongulense* strains is shown in Table 1. Briefly, the genome size ranged from 19.9 Mb to 20.1 Mb, the GC content ranged from 49.1 to 49.2%, and the BUSCO score was 92.4 to 92.7%. Consequently, the number of protein coding genes predicted ranged from 7,183 to 7,250.

**TABLE 1:** Assembly summary for draft genomic data of five Cystobasidium ongulense strains

	Data for strain:				
Characteristic	9A-2 (=JCM 31527)	9A-5 (=JCM 31528)	051-20-1	056-1-20Y-3	056-20-8
Sampling site	East Ongule Island	East Ongule Island	Inhovde	Inhovde	Inhovde
No. of reads	8,304,954,096	6,269,707,240	7,317,973,400	7,758.637,304	7,164,512,704
No. of scaffolds	69	67	120	73	81
Total length (Mb)	19.9	19.9	20.1	20.0	20.0
Coverage (×)	392	297	337	361	330
GC content (%)	49.2	49.2	49.2	49.1	49.1
Scaffold *N*_50_ value (kb)	2,125	2,126	1,057	1,432	1,222
No. of proteins	7,190	7,183	7,243	7,227	7,250
BUSCO score (%)	92.6	92.6	92.7	92.6	92.4
GenBank accession no.	BQUO00000000.1	BQUP00000000.1	BQUQ00000000.1	BQUR00000000.1	BQUS00000000.1
SRA accession no.	DRR118811, DRR118812	DRR118813, DRR118814	DRR118815, DRR118816	DRR118817, DRR118818	DRR118819, DRR118820

We believe that the genomic information of *C. ongulense* will help us understand how Antarctic fungi adapt to the extreme environment.

### Data availability.

This whole-genome shotgun project has been deposited at DDBJ/EMBL/GenBank under BioProject accession number PRJDB6568 with accession numbers BQUO00000000.1 (9A-2), BQUP00000000.1 (9A-5), BQUQ00000000.1 (051-20-1), BQUR00000000.1 (056-1-20Y-3), and BQUS00000000.1 (056-20-8). The raw reads are available under SRA accession numbers DRR118811, DRR118812 (9A-2), DRR118813, DRR118814 (9A-5), DRR118815, DRR118816 (051-20-1), DRR118817, DRR118818 (056-1-20Y-3), and DRR118819, DRR118820 (056-20-8).
